# Graph analysis of cell clusters forming vascular networks

**DOI:** 10.1098/rsos.171592

**Published:** 2018-03-14

**Authors:** A. P. Alves, O. N. Mesquita, J. Gómez-Gardeñes, U. Agero

**Affiliations:** 1 Departamento de Física, Universidade Federal de Minas Gerais- 31270-901 Belo Horizonte, MG, Brazil; 2 Departamento de Física de la Materia Condensada, Universidad de Zaragoza, 50009 Zaragoza, Spain; 3 GOTHAM Lab, Institute for Biocomputation and Physics of Complex Systems (BIFI), University of Zaragoza, 50018 Zaragoza, Spain

**Keywords:** vasculogenesis, complex networks, anti-angiogenic regulators, percolation

## Abstract

This manuscript describes the experimental observation of vasculogenesis in chick embryos by means of network analysis. The formation of the vascular network was observed in the *area opaca* of embryos from 40 to 55 h of development. In the *area opaca* endothelial cell clusters self-organize as a primitive and approximately regular network of capillaries. The process was observed by bright-field microscopy in control embryos and in embryos treated with Bevacizumab (Avastin^®^), an antibody that inhibits the signalling of the vascular endothelial growth factor (VEGF). The sequence of images of the vascular growth were thresholded, and used to quantify the forming network in control and Avastin-treated embryos. This characterization is made by measuring vessels density, number of cell clusters and the largest cluster density. From the original images, the topology of the vascular network was extracted and characterized by means of the usual network metrics such as: the degree distribution, average clustering coefficient, average short path length and assortativity, among others. This analysis allows to monitor how the largest connected cluster of the vascular network evolves in time and provides with quantitative evidence of the disruptive effects that Avastin has on the tree structure of vascular networks.

## 1. Introduction

Vasculogenesis is the process of de novo blood vessel formation [1,2]. At early stages of development, a forming vascular network involves several processes such as migration, proliferation and aggregation of endothelial cells (ECs) [1,3]. In this process, the vascular endothelial growth factor (VEGF) is the principal regulator of EC dynamics [4]. In recent years, spurred by the potential application to therapies aimed to reduce tumour growth rate, a large body of mathematical models have been developed to better understand vessel formation [2,5–10]. Several studies have shown that cultures of EC aggregates coalesce forming a connected vascular network [7,8,11] so that, at a critical cell density, a percolation transition was observed [8,12]. Although the mechanisms involved in vasculogenesis have been largely studied using EC assembly [7–10,13], we still lack quantitative analysis to understand how a vascular network is formed in a more complex system [14,15]. In this regard, the development of new visualization techniques has allowed to monitor the vasculogenesis process on a whole embryo [2,15,16]. In parallel to the studies aimed at understanding vasculogenesis, a large effort has been made to find anti-angiogenic regulators that control vessel density preventing tumour nourishment [17–19]. In particular, it has been shown that a low dose of Bevacizumab (Avastin^®^) administered in tumour regions induces the renormalization of the vascular network, improving the oxygenation and drug penetration into tumours. As a consequence, the anti-angiogenic factor slows down the tumour dissemination and enhances the effectiveness of chemo- and radiotherapies [19].

The goal of this work is twofold. First, we have performed experiments by means of bright-field video-microscopy in order to monitor the vasculogenesis process in chicken embryos during the first 15 h of development. These experiments allow us to obtain quantitative data about vessel formation through a systematic characterization of the vascular network evolution from its onset on control and on Avastin-treated embryos. The quantitative data include vessel density, number of cell clusters and the largest cluster density measured as a function of time. Second, taking advantage of the experimental parameters derived by monitoring vasculogenesis, we extract the topology of the forming vascular network and measure the effect of Avastin on its morphology. The quantitative comparison between the structural properties of vascular network on control and Avastin-treated embryos is carried out by measuring network topological features [20] and monitoring its evolution along the vasculogenesis process. The results obtained allow to better understand how EC aggregates self-organized to form a connected vascular network and quantify the morphological differences between the vascular networks of control and Avastin-treated groups.

## 2. Material and methods

### 2.1. Experimental set-up

**Figure 1.**
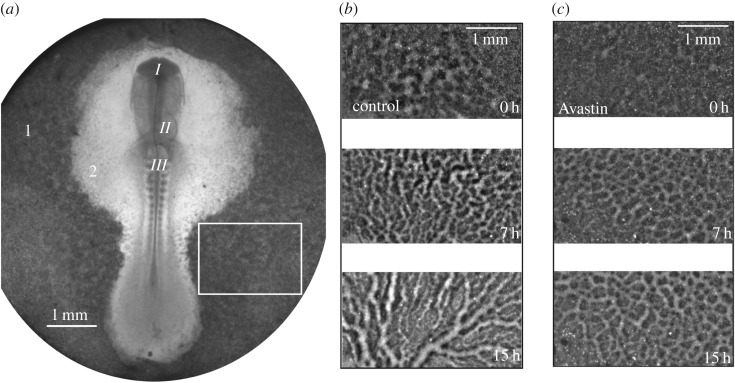
(*a*) Whole embryo image. From top to bottom, around the middle line, we see the head fold *I*, forming heart *II* and somites *III*, which are blocks of cells which will give rise to muscles, bone, cartilage and skin. The number 1 indicates the *area opaca* and 2 is the *area pellucida*. A selected rectangle (6.58 mm^2^) in the *area opaca* is used to monitor the forming vascular network. A zoom of the rectangular area was taken from (*b*) a control embryo, (*c*) an Avastin-treated embryo, at times: 0, 7 and 15 h, from top to bottom.

The first cell clusters emerge in the *area opaca* after nearly 40 h of incubation. At this time, the whole embryo was extracted and put under a bright-field microscope to follow vessel formation. Bright-field microscopy is the simplest and cheaper optical microscopy technique. We used it because the contrast obtained was good enough for the imaging analysis we have performed. The complete description of embryo preparation is presented in [21,22]. An image of a whole embryo before formation of vessels at Hamburger and Hamilton (HH) stage 11 [23] is shown in figure 1*a*, showing that, at this stage, the chicken embryo is a two-dimensional small disc. We then monitor the emergence of vessels in the *area opaca* from stage HH-11 to HH-16 [23], i.e. comprising 15 h of development. The original movies can be found in the electronic supplementary material; *VesselGrowth-ControlEmbryo.avi* and *VesselGrowth-TreatedEmbryo.avi* show the formation of vessels on control and Avastin-treated embryos. In figure 1, we show three snapshots of these movies corresponding to 0 h, 7 h and 15 h for control (figure 1*b*) and Avastin-treated (figure 1*c*) embryos in a region (6.58 mm^2^) within the *area opaca*. Avastin is incorporated by adding 30 μl at a concentration of 6 μg μl^−1^ just after the embryo is extracted. The movies were captured using a Nikon Eclipse TI inverted microscope equipped with an objective 1X Nikon Plan UW NA 0.04 and WD 3.2 mm (Nikon Part # MRL 00012) and an environmental chamber (model Chamlide IC-CU:109, Live Cell Instrument) which provides the environment at 5% CO_2_ atmosphere, 37^°^C temperature and 60% humidity. Finally, the images were captured using a 12 bit Uniq camera (model 1800 CL, Epix Inc.) with 4096 grey levels, 6.4 μm of pixel square side, a gain of 11.04 dB and a time resolution of one image captured every 17.28 s.

### 2.2. Image processing

**Figure 2.**
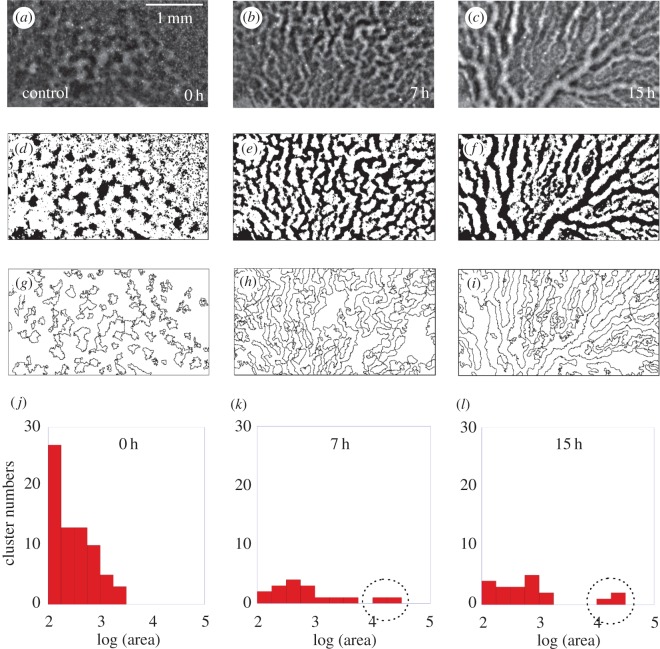
Original images (*a*–*c*). Binary images (*d*–*f*), after a threshold operation show an equivalent pattern when compared with the original images. Contour lines of clusters, (*g*–*i*), identified by the plug-in *Analyze Particles*. From the processed images, we measure the number of endothelial cell clusters and their area. Area distribution of endothelial cell clusters (*j*–*l*) from the image sequence (*d*–*f*). (*j*) Displays the distribution at the initial stage. In the middle of the time-interval analysed the distribution is represented in (*k*), where a large cluster starts to appear as identified by the dotted circle.

Vessel patterns were extracted by means of the plug-in *Analyze Particles* from *ImageJ* [24]. To use this plug-in, a grey level threshold was carefully chosen for each embryo to keep as much information as possible about the contours of the original images. To illustrate this process, we show in figure 2*a*–*c* three real pictures captured (already shown in figure 1*b*) and below, in figure 2*d*–*f*, we show their corresponding version once the threshold has been applied. The resulting binary images are then used to identify the EC clusters, as shown in figure 2*g*–*i*. Binary images were also used to measure fractal dimension on binary images applying the box counting method using the plug-in *FracLac* [25], also from *ImageJ* [24]. We used a box size ranging from 2 to 200 pixels following a power series to scan rectangular images with an area of 6.58 μm^2^. These techniques allow us to analyse the spatio-temporal self-organization of EC clusters forming the vascular network. For instance, in figure 2*j*–*l*, we show the area distribution of the EC clusters of images in figure 2*a*–*c* obtained for control embryos. Each distribution displays the number of cell clusters as a function of its area (those clusters smaller than 1.2×10^−4^ mm^2^ were excluded). From the evolution of the area distribution, it is clear that a large cluster (marked by the dotted circle) emerges as time increases.

### 2.3. Network analysis

**Figure 3.**
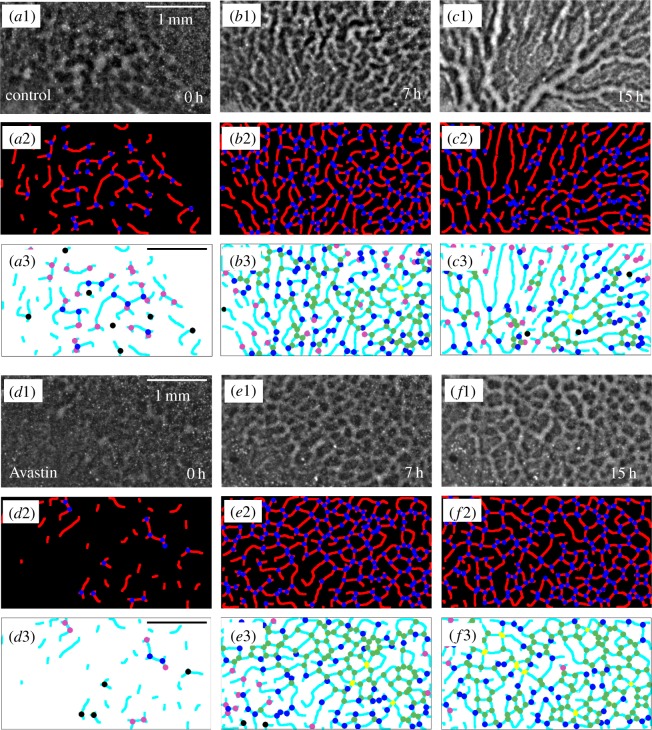
Original images (*a*1–*c*1), (*d*1–*f*1) for a control and an Avastin-treated embryo, respectively. Network topology (*a*2–*c*2), (*d*2–*f*2) obtained using the program AngioTool shows an equivalent pattern when compared with the original images. The node degree (*a*3–*c*3 and *d*3–*f*3) where each colour represents the node degree or its absence (*black*=*without* link, *pink*=*one* link, *blue*=*two* links, *green*=*three* links, *yellow*=*four* links).

Vascular networks are constructed, as usual in spatial networks formed by roads and intersections [26], by considering the vessels as the links and the bifurcation points as representing the nodes of the network. The topology of the network is obtained by means of the free software AngioTool, developed to extract the quantitative analysis of angiogenesis [27]. This software is applied directly on original images to identify the vessels for a range of diameters and intensities previously selected. In figure 3(*a*2–*c*2) and (*d*2–*f*2), we show how the two-dimensional network topology is extracted from the original image sequences (*a*1–*c*1) and (*d*1–*f*1) for a control and an Avastin-treated embryo, respectively. The resulting networks are represented in figure 3(*a*3–*c*3) and (*d*3–*f*3), where the colours of nodes represent the number of neighbours (the degree) particular to each node. Once the network is constructed, and in order to apply graph-theoretic tools, we encode its topology into an *N*×*N* (*N* being the number of nodes) adjacency matrix, **A**, whose elements are: *A*_*ij*_=1 if nodes *i* and *j* are connected and *A*_*ij*_=0 otherwise. This way the degree of a node *i* is

2.1 $$k_{i}=\sum_{j=1}^{N}A_{ij}.$$

In what follows, we present a brief summary of the graph metrics [26,28,29] implemented to characterize the networks obtained during the vasculogenesis process:

— *Degree distribution*, *P*(*k*) is the probability that a node chosen at random has *k* neighbours. This distribution is computed by measuring the number of nodes *N*(*k*) with *k* neighbours divided by the total number of nodes, *N*:
  2.2 $$P(k)=\frac{1}{N}\sum_{k^{\prime }=k}^{\infty }N(k^{\prime }).$$
— The average degree 〈*k*〉 of a network is the first moment of its degree distribution *P*(*k*) and can be calculated as
  2.3 $$\left\langle k\right\rangle =\frac{1}{N}\sum_{i=1}^{N}k_{i}=\frac{1}{N}\sum_{i=1}^{N}\sum_{j=1}^{N}A_{ij}.$$
— The gamma index *γ* measures the connectivity in a network. It is defined as the ratio of the number of links in the network, *n*_ℓ_, to the maximum possible number of links between nodes that, in a planar graph, is equal to 3(*N*−2). Therefore, the expression of the *γ* index reads
  2.4 $$\gamma =\frac{n_{\ell }}{3(N-2)}.$$
  The value of gamma lies in the range *γ*∈[0,1], where *γ*=0 represents a case in which there are no connections between nodes, whereas *γ*=1 correspond to maximally connected network [29,26].
— The average shortest path length of a network 〈*L*〉 measures the characteristic distance between each pair of nodes in the network [26,28,30]. The distance *d*_*ij*_ between two nodes *i* and *j* is computed as the minimum number of links that has to be crossed to go from *i* to *j*. By considering all the couples in the network, *L* can be written as
  2.5 $$\langle L\rangle =\frac{1}{N(N-1)}\sum_{i\neq j}d_{ij}.$$
— Treeness *ϕ* is used to measure the hierarchical organization on network topologies [29]. A tree graph can be described as a set of connected nodes without closed paths. The treeness covers the ranges from non-hierarchical, *ϕ*=0 (the graph displays all closed paths), to hierarchical, *ϕ*=1 (the graph displays a tree-like structure) which is defined by the following equation:
  2.6 $$\phi =\frac{L_{\mathrm{Tree}}}{\left\langle L\right\rangle },$$
  where *L*_Tree_ is the sum of the average path length of the graph without closed circuits and 〈*L*〉 is the average path length of the entire graph.
— The clustering coefficient 〈C〉 measures the cohesiveness of the network. It is measured as the average of all local (nodal) clustering coefficients in the network divided by the total number of nodes *N*. The local clustering of a node *i* is the ratio between the number of links among the neighbours connected to *i*, *e*_*i*_, and the maximum possible number of links between them: *k*_*i*_(*k*_*i*_−1)/2:
  2.7 $$\langle C\rangle =\frac{1}{N}\sum_{i}^{N}\frac{2e_{i}}{k_{i}(k_{i}-1)}.$$
  By the definition, the value is in the interval of 0≤〈C〉≤1 [30,28].
— The assortativity coefficient *r* measures the tendency of nodes with similar degree to be connected [31,32] and can be written as
  2.8 $$r=\frac{\langle k_{i}k_{j}\rangle -{\left\langle k\right\rangle }^{2}}{\langle k^{2}\rangle -{\left\langle k\right\rangle }^{2}},$$
  where 〈*k*_*i*_*k*_*j*_〉 is the average of the products of the degrees of nodes that are connected to each other, 〈*k*〉^2^ is the square of the mean node degree and 〈*k*^2^〉 is the second moment of the degree distribution *P*(*k*).

## 3. Results and discussion

We organize the results assembling the parameters that better characterize the stages of the network formation as follows.

(a) Early network formation: involves migration and aggregation among EC clusters, which is characterized through *Statistical analysis of binary patterns*.
(b) Network formation: identified by a percolation phase transition in the system, which can be determined by *Network metrics*, some parameters display an abrupt transition near 5 h.
(c) Network remodelling: differentiation of vascular network, which will be identified by *Structural metrics* parameters measured on network topologies. We were able to distinguish tree-like topology from the more lattice-like structure near 7.8 h.

### 3.1. Statistical analysis of binary patterns

We now proceed to show the main results that characterize the spatio-temporal self-organization of EC clusters forming the vascular network in both groups of control and Avastin-treated chick embryos. As anticipated above, in figure 2*j*–*l* we show the area distribution at three different stages. In the initial stage, cell clusters are still small; in the middle of the process, the area distribution in figure 2*k* points out many small clusters while a largest cluster appears. At the final stage, the largest cluster is clearly far apart from the distribution of small clusters, as shown in figure 2*l*. The area of the largest cluster reaches a maximum value of *A*_Largest Cluster_=1.7±0.2 mm^2^, which is three orders of magnitude larger than the area of the initial clusters. The movies provided in the electronic supplementary material show the vessels’ growth within the *area opaca* for both control and Avastin-treated embryos over 15 h of the vasculogenesis process.

**Figure 4.**
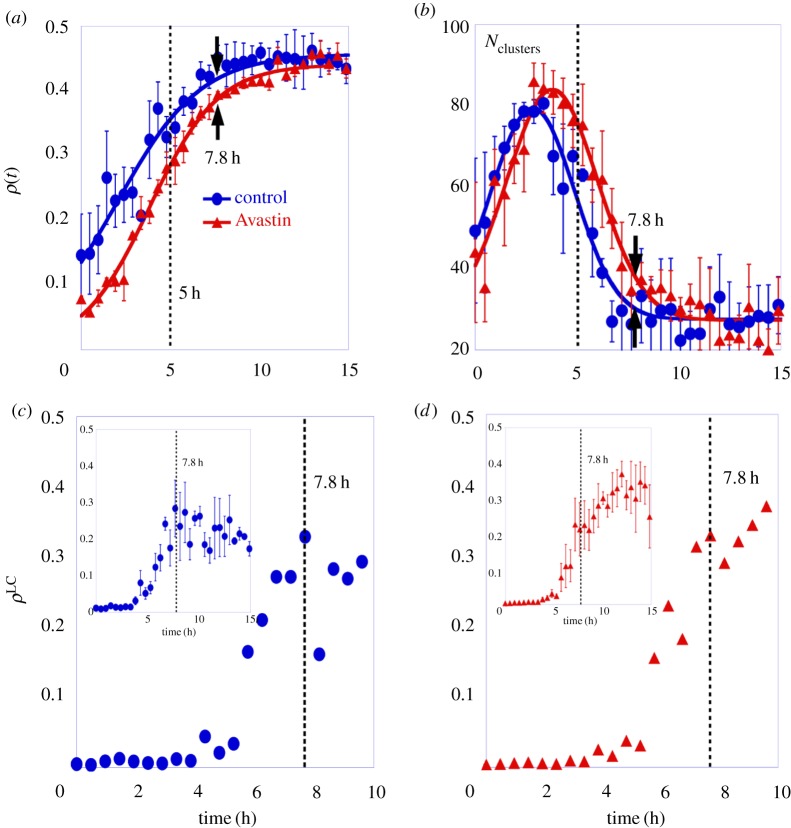
Statistical analysis of binary patterns which characterize the spatio-temporal self-organization of cell clusters forming the vascular network for three embryos in each group. The error bars are the standard deviation. (*a*) Mean vessel density of endothelial cell clusters, *ρ*(*t*), as a function of time; the full lines are the fit obtained from equation (3.1). (*b*) The mean number of cell clusters, *N*_*clusters*_, as a function of time, where the full line is a Gaussian fit just to guide the eye. The largest cluster density, *ρ*^*LC*^, as a function of time for (*c*) one control embryo and (*d*) one Avastin-treated embryo. The insets display the average of the largest cluster density as a function of time for both groups. The vertical line is a guide to the eye for two characteristic times at 5 h in (*a*) and (*b*), 7.8 h in (*c*) and (*d*). The full circles represent the data for control, and the full triangles the data for Avastin-treated embryos.

Figure 4*a* shows the mean vessel density *ρ*(*t*), for control and for Avastin-treated embryos as a function of time. This quantity is defined as *ρ*(*t*)=(detected vessel area)/(total image area), and it is fitted by equation (3.1).

To characterize vessel growth for both groups, we use a nonlinear equation as a model which displays a similar behaviour of non-equilibrium phase transition models [33,34]. We fitted the experimental data shown in figure 4 using the solution of a logistic growth equation, as follows:

3.1 $$\rho (t)=\frac{1}{\left[1/\rho_{\max}+(1/\rho_{0}-1/\rho_{\max})\;e^{-\omega t}\right]},$$

where *ρ*(*t*) is the vessel density, *ρ*_0_ is the initial vessel density, *ρ*_max_ is the maximum density value and *ω* is the growth rate. The maximum density value reached for control, *ρ*_max_^Control^=0.46±0.01, is close to the maximum density obtained for the treated embryos, *ρ*_max_^Avastin^=0.44±0.01. It shows that although the Avastin concentration used in this work decreases vessels density, is not enough to inhibit the vessel formation. The vessel density growth rate is *ω*^Control^=(0.42±0.04)/*h* for a control group and *ω*^Avastin^=(0.52±0.02)/*h* for Avastin-treated group. The saturation *plateau* obtained on treated embryos is a little lower than the one reached for control ones.

Figure 4*b* shows the time evolution of the mean number of cell clusters, *N*_clusters_, for a group of control and for a group of Avastin-treated embryos. In control embryos the value of *N*_clusters_ increases reaching a maximum after 2.7 h, then it decreases to a minimum value when the vascular network is completely assembled. On the other hand, for Avastin-treated embryos *N*_clusters_ shows the same qualitative behaviour, although it reaches a maximum next to 3.5 h. The *N*_clusters_ measured at final stages are the smallest value measured, indicating that the EC clusters aggregate giving rise to a largest cluster on *area opaca*.

The relative size of the largest interconnected cluster has been measured to characterize the percolation phase transition on vasculogenesis as shown in [35,8]. The largest cluster density, *ρ*^LC^, is defined as the ratio between the area of the largest interconnected cluster formed by aggregation of cell clusters, divided by the total area. To show the emergence of the percolating cluster on it the system, figure 4*c* shows the largest cluster density for one control embryo in an interval of 10 h, and the inset shows the average in 15 h for a group of control embryos. The same data are shown in figure 4*d* for one treated embryo in a interval of 10 h, which starts with a small value and undergoes an abrupt change after 5 h, reaching a maximum value (*ρ*^LC^≈0.3). And the inset shows the average in 15 h for a group of Avastin-treated embryos. At an interval of 10 h, the largest cluster density is approximately equal to that of the system, indicating that a percolating cluster is formed, as described in [36]. In this work, the largest cluster density is the first measurement which points out the abrupt phase transition on the vasculogenesis process identifying two important times, one at 5 h, where the *ρ*^LC^ starts to grow, and the other at 7.8 h where it reaches a saturation value. A percolation transition has been previously observed analysing ECs in culture, as reported in [35,8].

### 3.2. Network metrics

**Figure 5.**
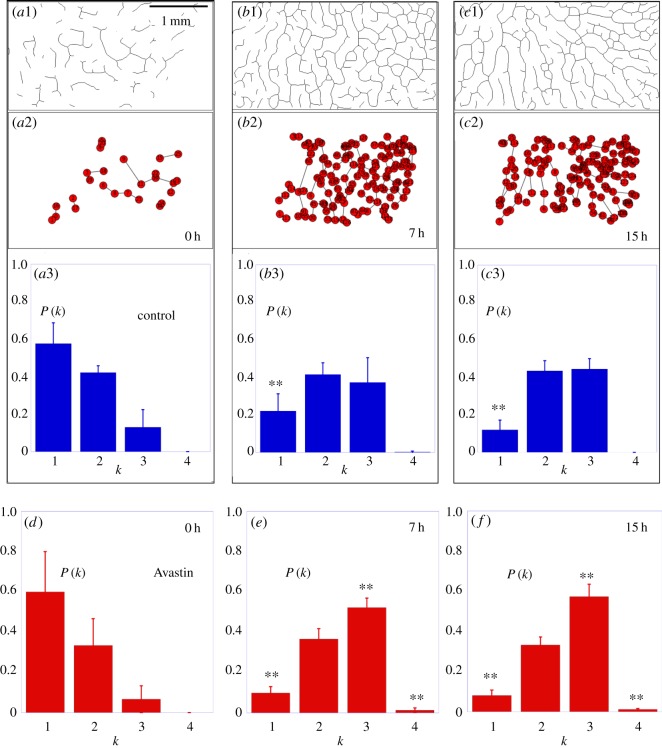
The codification process of adjacent matrix: (*a*1–*c*1) show the skeleton topology of a forming vascular network for a control embryo and (*a*2–*c*2) represent the adjacent matrix extracted from the skeleton, where each bifurcation is identified as a node, and the distance interaction represents the links. The node degree distributions obtained from three embryos in each group as displayed for (*a*3–*c*3) the control and (*d*–*f*) Avastin-treated group. The symbol (**) indicates the statistical difference of the distributions from the initial stage at *t*=0 *h*, which have *p*<0.05 calculated by the Smirnov–Kolmogorov test. The error bars are the standard deviations.

Now we focus our attention to characterize the structural properties by means of *Network metrics* [26,28,29] of vascular network topology as introduced in figure 3(*a*2–*c*2) and (*d*2–*f*2). The structure of these networks will reveal an important percolation transition in the system. Figure 5(*a*1–*c*1) shows the skeleton of a forming vascular network in which we extracted the adjacent matrix that encodes the structure of the graphs plotted in figure 5(*a*2–*c*2). We start by showing the time evolution of the degree distribution *P*(*k*) for a group of control and Avastin-treated embryos (see figure 5(*a*3–*c*3) and (*d*–*f*), respectively). The planar vascular networks contain nodes with few neighbours, as the degrees of the vertices range from 1 to 4. At the initial stage, 0 h, on control group ≈60% of nodes have degree 1, ≈40% have degree 2, ≈15% degree 3 and nodes with degree 4 are not observed. Once a large connected cluster has been formed, to 7 h, we find that just ≈20% of nodes have degree 1, ≈37% have degree 2, ≈40% have degree 3, and just ≈2% of nodes have degree 4. At the final stage, we observe that ≈20% of nodes have degree 1, ≈40% have degree 2 and 3, and nodes with degree 4 are not observed as shown on figure 5(*a*3–*c*3). The evolution of the degree distribution *P*(*k*) shows the maturation of the vascular networks during the vasculogenesis process. On the other hand, although a similar behaviour is observed for the evolution of *P*(*k*) in the Avastin-treated group, in this case the fraction of nodes with degree 3 is significatively larger at the final stage, reaching to ≈60% of nodes in the final stage. This increase is achieved at the expense of a decrease in the fraction of nodes connected to two neighbours.

**Figure 6.**
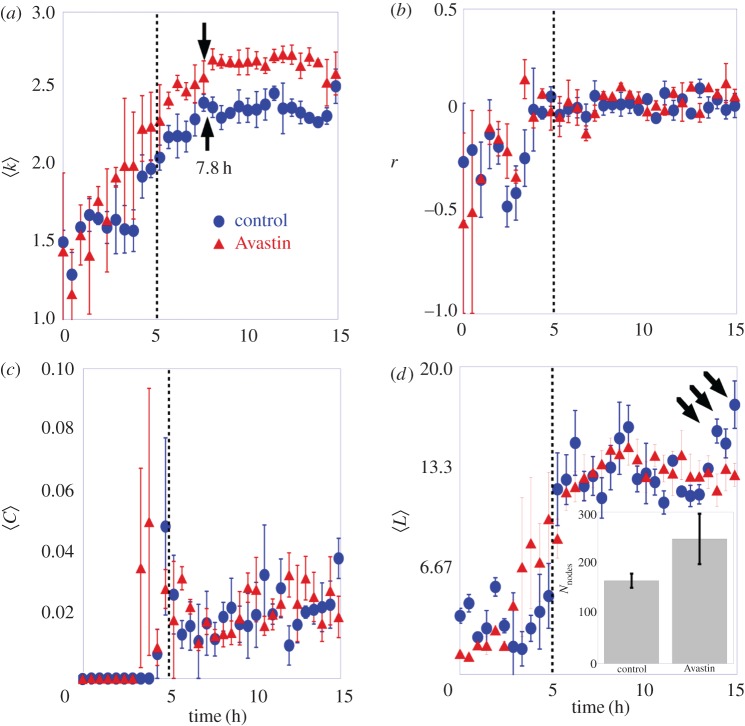
Network descriptors of vascular network topologies. The filled circles represent the group of control, and the filled triangles a group of Avastin-treated embryos. (*a*) The mean node degree as a function of time measured on a complete vascular network. (*b*) The coefficient of assortativity measured on the largest cluster as a function of time. (*c*) The average clustering coefficient as a function of time measured on a complete vascular network. (*d*) The shortest path length measured on the largest cluster as a function of time. The error bar is the standard deviation for three embryos in each group. The black arrows highlight the larger value reached by the control group at the final stage. The inset graph shows the average number of nodes with the respective error bar for both groups. The vertical line is a guide to the eye for the time of 5 h.

The more densely connected structure of vascular networks in the Avastin-treated group is also confirmed by monitoring the evolution of the average degree 〈*k*〉 during vasculogenesis. In figure 6*a*, we show this evolution for control and Avastin-treated embryos. It becomes clear that, once the network has been formed, the average degree increases and reaches a *plateau* close to 〈*k*_control_〉=2.28±0.06 on control, and 〈*k*_Avastin_〉=2.54±0.05 on Avastin-treated embryos. The average degree tends to remain constant after 7.8 h as identified by the black arrows in figure 6*a*, pointing out that the vascular network has been completely formed.

We now monitor the evolution of other network metrics during vasculogenesis. In figure 6*b*, we show the time evolution of the assortativity coefficient *r* for control and Avastin-treated embryos. Initially, the assortativity coefficient is negative *r*<0 for both groups, pointing out that networks are disassortative at this initial stage due to the formation of many small star-like structures before the large connected cluster shows up. When this happens, assortativity becomes nearly zero, *r*∼0, indicating that the degrees of connected nodes tend to be uncorrelated due to their similarity. This evolution holds for control and Avastin-treated embryos because both for tree-like (control embryos) and lattice-like (Avastin-treated embryos) structures the degrees of adjacent nodes are similar. In between the assortativity oscillates, with a minimum near the same time we observe the maximum number of clusters, as shown in figure 4*b*. The creation of new small star-like clusters compete with the large emerging cluster which contributes to abruptly decreasing the assortativity coefficient around the time of 3 h.

The evolution of the clustering coefficient 〈C〉 of the networks of control and Avastin-treated embryos is shown in figure 6*c*. At the initial stages of development, clustering is very small, as it obviously follows from the abundance of star-like structures in the early vascular network. However, a sudden growth takes place both for control and Avastin-treated embryos at *t*≃5 *h*. After this jump the clustering reaches a *plateu*, stabilizing around a small value 〈C〉≈0.021±0.007 for both control and Avastin-treated groups, which is consistent with the tree-like and lattice structures that appear in their respective developed networks.

The time evolution of the average shortest path length, 〈*L*〉, is shown in figure 6*d*, while the inset shows the mean number of nodes on control and Avastin-treated groups. We computed 〈*L*〉 of the largest connected cluster in order to avoid its divergence due to the presence of disconnected nodes. The evolution of 〈*L*〉(*t*) shows a sharp increase at *t*≃5 *h*, reaching a stationary value close to 〈*L*〉≈13 for both control and Avastin-treated groups. However, in the last hours (see black arrows) 〈*L*〉 increases on control group. Although the value of 〈*L*〉 is very close for both groups, the larger number of nodes in the Avastin-treated embryos (see inset) is a signature that the connectivity of its vascular network is larger.

**Figure 7.**
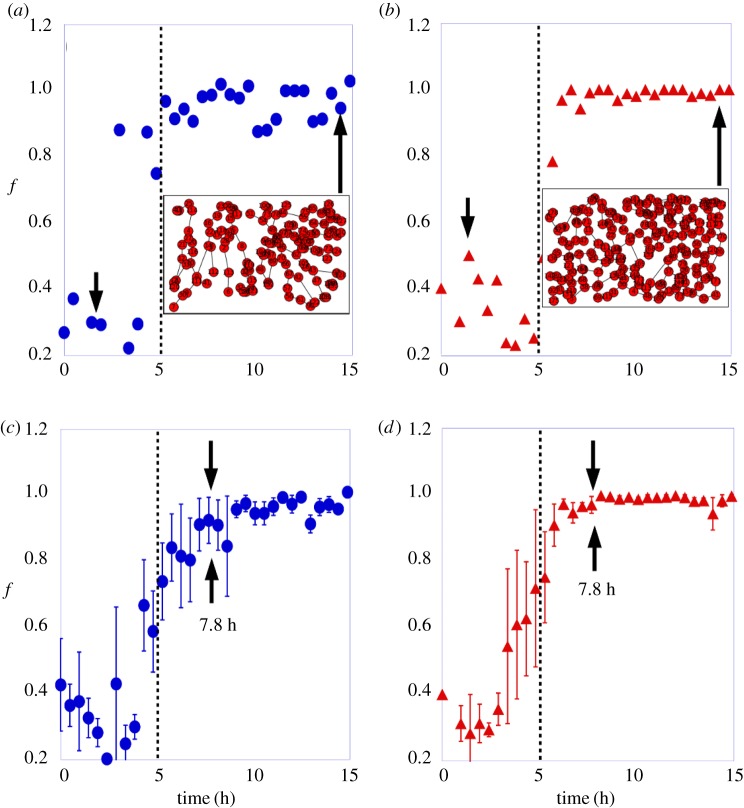
The fraction of nodes in the largest cluster, *f*, as a function of time for (*a*) one control sample, and (*b*) for one Avastin-treated embryo. The insets display the spatial position of vascular network bifurcation. The *f* as a function of time in a group of three embryos for (*c*) control, and (*d*) Avastin-treated ones. The error bar is the standard deviation. The filled circles represent the data for control, the filled triangles the data for Avastin-treated embryos, and the vertical dotted line is a guide to the eye for the time of 5 h.

Finally, in figure 7*a*,*b,* we show the fraction of nodes on the largest cluster, *f*, as a function of time in one control and one Avastin-treated embryo, respectively. The insets in the plots show the particular vascular networks at the final stage. The fraction of nodes on the largest cluster starts with small values and abruptly grows at approximately 5 h on control and Avastin-treated embryos reaching soon afterwards its maximum possible value *f*=1. This abrupt transition from small to larger values is clear when just one sample is monitored. In figure 7*c*,*d*, we show the evolution of *f* averaged in a group of control and Avastin-treated embryos. In this case, the transition becomes smoother although the jump at approximately 5 h is still clear.

### 3.3. Structural metrics

To point out the structural change promoted by Avastin in the vascular network morphology we measured: the chord length ℓ (mm), the fractal dimension *D*_f_, the treeness *ϕ* and the *γ* index.

**Figure 8.**
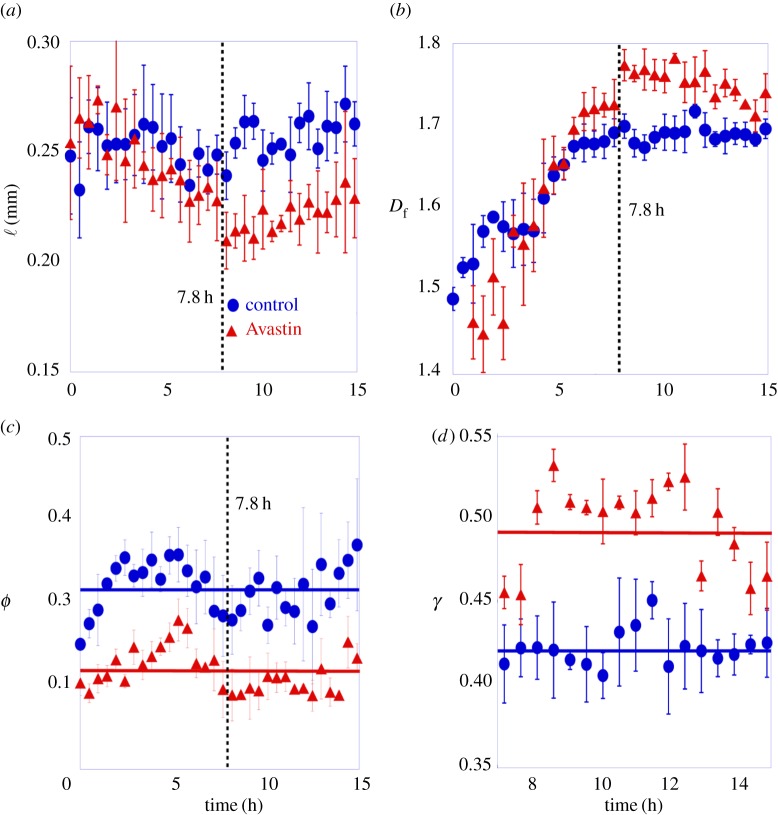
Structural metrics measured on network topologies. (*a*) The mean cord length, ℓ, (*b*) the mean fractal dimension measured on a forming vascular network, *D*_f_, (*c*) treeness, *φ* and (*d*) the *γ* index measured on a formed vascular network. The error bar is the standard deviation of each group, compound by three samples. The full lines in (*c*) and (*d*) indicate the mean value of the indexes. The vertical line is a guide to the eye for the time of 7.8 h.

Figure 8*a* shows the mean chord length ℓ (mm), which represents the distance between vessel bifurcations as a function of time, for a group of control and Avastin-treated embryos. At the initial stages, the mean chord length measured on both control and Avastin-treated embryos is ℓ=0.25±0.03 mm. After approximately 7.8 h of the experiment, we observe in figure 8*a* that the mean chord length splits out in two distinct values, reaching a mean value of ℓ^Control^=0.26±0.01 mm on control, and a mean value of ℓ^Avastin^=0.22±0.01 mm on Avastin-treated embryos. The smaller value measured on Avastin-treated embryos indicates that Avastin interacts on the system promoting structural changes on the vascular network. The mean chord length obtained on the treated group is near to the value measured for ECs randomly spread on a matrigel ℓ^ECMatrigel^=0.20±0.02 mm as reported in [13,8].

Figure 8*b* shows the evolution of the mean fractal dimension over time for control and for Avastin-treated embryos. The fractal dimension measured during the initial stages in control is *D*_f_=1.49±0.01 while in Avastin-treated embryos is *D*_f_=1.44±0.04. These values are close to the fractal dimension measured on *cluster–cluster aggregation*, which presents *D*_f_≈1.5 as shown in [37,12]. It suggests that, in the first hours of development, cluster–cluster aggregation prevails on the growth dynamics and new clusters emerge on the *area opaca* as shown in figure 4*b*. The fractal dimension increases during the first hours. After 5 h of experiment the mean fractal dimension measured for both groups splits out in two different values near 7.8 h and reaches *a plateau* as shown in figure 8*b*. The mean fractal dimension measured from 5 to 15 h is *D*_f_=1.69±0.01 in the control group and *D*_f_=1.74±0.02 in the Avastin-treated group. A higher fractal dimension measured on the Avastin-treated group indicates that this group presents a large uniform vessel distribution; a similar behaviour has been reported for vascular tissue as shown in [38–40].

The former result corroborates that in the Avastin-treated group the tree structure is broken and, consequently, the number of connections on the vascular network grows. To get more insight on this effect of Avastin, we have measured the treeness *ϕ* (equation (2.6)) to characterize the evolution of the ramification in vascular networks. In figure 8*c*, we plot the time evolution of *ϕ* for control and Avastin-treated embryos. At the initial stage, the treeness of both groups displays similar values (*ϕ*∼0.1). As the network evolves, treeness increases considerably for the control and Avastin-treated embryos and, close to 7.8 h (dotted line), it decreases in both groups to finally stabilize around *ϕ*_Control_=0.39±0.04 on control and *ϕ*_Avastin_=0.19±0.09 on Avastin-treated embryos. The larger stationary treeness for the control group suggests that their vascular networks resemble more a tree-like topology while, in this regard, the action of Avastin promotes more densely connected regions, thus losing the branched architecture of control embryos and displaying an architecture that resembles that of urban-like structures [29]. This effect of Avastin may play an important role against tumour growth. To further validate these findings, we have monitored the evolution of vascular networks in terms of their *γ* index (equation (2.4)).The evolution of *γ*, shown in figure 8*d*, indicates that the density of links in Avastin-treated networks is larger than that found in the networks of control embryos. All the results shown in figure 8 corroborate the described structural changes promoted by Avastin and go in the same line as some previous reports made in [19].

## 4. Conclusion

In this article, we characterize the spatio-temporal self-organization of EC clusters forming vascular networks on control and on Avastin-treated embryos. To this aim, we monitor how EC clusters aggregate to form a connected vascular network and pinpoint the main differences between the two groups through statistical and network analysis. In a nutshell, we have observed that while the percolation transition of control networks yields a typical tree-like structure, the anti-angiogenic factor makes the resulting networks more lattice-like. This observation is supported by different evidence when monitoring the time evolution of metrics such as: (i) the mean chord length, (ii) the fractal dimension of networks *D*_f_, (iii) the degree distribution *P*(*k*), (iv) the mean degree 〈*k*〉, (v) the *γ*-index and (vi) the treeness *ϕ*; some of these parameters display a discrepancy near 7.8 h, evidencing the structural change in the network topology. On the other hand, other measures such as (i) the clustering coefficient C, (ii) the network assortativity *r*, (iii) the average path length 〈*L*〉 and (iv) the fraction of nodes in the largest cluster f, do not display differences between control and Avastin-treated embryos, because tree-like and lattice-like structures display similar values for these quantities. Also importantly, we have shown for both groups that, after approximately 5 h, some of these parameters point to an abrupt transition from disconnected cell clusters to a large connected vascular network which is a signature of the percolation in the system.

These results attest that an optimal dose of an anti-angiogenic factor VEGF on a normal embryo shapes the resulting vascular network. The new graph structure could be more prone to drug circulation and penetration than the usual vascular architecture. Apart from these results, we believe that the methodology used in this study can be easily generalized to evaluate the goodness of different anti-angiogenic therapies based on network metrics that allow quantitative comparisons. On more general grounds, the mechanisms governing the vascular network formation may be universal and similar analysis in other species should give similar results.

## Data accessibility

To give support for the data present in this work, there are two movies which show the growth of vessels within the *area opaca* for control and for Avastin-treated embryos. *VesselGrowth-ControlEmbryo.avi* is a movie which shows the self-organization of endothelial cell clusters forming the vascular network within the *area opaca* for an embryo without drugs. *VesselGrowth-TreatedEmbryo.avi* is a movie which shows the self-organization of endothelial cell clusters forming the vascular network within the *area opaca* for an embryo treated with Avastin.
